# Novel Hybrid Conductor of Irregularly Patterned Graphene Mesh and Silver Nanowire Networks

**DOI:** 10.3390/mi11060578

**Published:** 2020-06-09

**Authors:** Hiesang Sohn, Weon Ho Shin, Dohyeong Seok, Taek Lee, Chulhwan Park, Jong-Min Oh, Se Yun Kim, Anusorn Seubsai

**Affiliations:** 1 Department of Chemical Engineering, Kwangwoon University, Seoul 01897, Korea; DohyeongSeok@kw.ac.kr (D.S.); tlee@kw.ac.kr (T.L.); chpark@kw.ac.kr (C.P.); 2 Department of Electronic Material Engineering, Kwangwoon University, Seoul 01897, Korea; weonho@kw.ac.kr (W.H.S.); jmOh@kw.ac.kr (J.-M.O.); 3 Material Research Center, Samsung Advanced Institute of Technology (SAIT), Samsung Electronics, Suwon 16678, Korea; seyuni.kim@samsung.com; 4 Department of Chemical Engineering, Faculty of Engineering, Kasetsart University, Bangkok 10900, Thailand; fengasn@ku.ac.th

**Keywords:** irregular patterning, graphene mesh, silver nanowire, hybrid conductor, chemical etching, flexible transparent conducting film, bending stability

## Abstract

We prepared the hybrid conductor of the Ag nanowire (NW) network and irregularly patterned graphene (GP) mesh with enhanced optical transmittance (~98.5%) and mechano-electric stability (Δ*R/R*_o_: ~42.4% at 200,000 (200k) cycles) under 6.7% strain. Irregularly patterned GP meshes were prepared with a bottom-side etching method using chemical etchant (HNO_3_). The GP mesh pattern was judiciously and easily tuned by the regulation of treatment time (0–180 min) and concentration (0–20 M) of chemical etchants. As-formed hybrid conductor of Ag NW and GP mesh exhibit enhanced/controllable electrical-optical properties and mechano-electric stabilities; hybrid conductor exhibits enhanced optical transmittance (*TT* = 98.5%) and improved conductivity (Δ*R_s_*: 22%) compared with that of a conventional hybrid conductor at similar *TT*. It is also noteworthy that our hybrid conductor shows far superior mechano-electric stability (Δ*R/R*_o_: ~42.4% at 200k cycles; *TT*: ~98.5%) to that of controls (Ag NW (Δ*R/R*_o_: ~293% at 200k cycles), Ag NW-pristine GP hybrid (Δ*R/R*_o_: ~121% at 200k cycles)) ascribed to our unique hybrid structure.

## 1. Introduction

Owing to growing demand of the modern electronic devices including smart phones, touch screens, and light-emitting displays, there have been massive demands on the transparent conductive electrodes (TCEs) and TCE materials (e.g., indium tin oxide (ITO)) [1,2,3,4]. Although it suffices for its job at the current technological stage, a combination of brittle nature, limited global supply of indium, and its high processing cost makes the conventional ITO based TCE unsuitable for next-generation applications such as flexible/stretchable displays and wearable devices. Therefore, there has been increasing drive to replace it with new classes of flexible TCE materials [1,2,3,4]. 

There have been reported many candidates for flexible TCE materials including metallic nanowires (e.g., Ag NW, Cu NW), graphene, carbon nanotube (CNT), and their hybrids (metal NWs-graphene and metal NWs-CNT) as TCE alternative to ITO for stretchable and flexible electronics [4,5,6,7,8,9,10,11]. For instance, graphene (GP)—the single atomic layer of carbon—has received attention as promising TCE material because of its superior mechanical strength and high conductivity [10,12,13]. Particularly, Ag NW-based conductors gained considerable interest owing to the low process cost as well as their mechanically durable flexibility and higher optical transparency than ITO at equivalent sheet resistance (*R*_s_) [4]. Despite the high stretchability of Ag NWs, however, the low intrinsic mechanical strength (4.8 GPa fracture strength for silver), low adhesion with other materials, and lack of functionalization methods hamper the production of a highly stretchable Ag NW-based TCE [4]. What is worse, Ag NW networks have a high junction resistance associated with charge transport between randomly oriented NWs which reduce the electrical conductivity of NW [4]. 

Ag NW-GP hybrid, thus, has received considerable attention as one of the most promising candidates for flexible TCE because of the complementary properties of Ag NW and GP [8,9,10,11]. Note that as-formed Ag NW network-GP hybrids exhibit high conductivity and mechanical robustness/flexibility through the inter-nanowire connection of Ag NW forged by GP. However, despite the potential of the Ag NW-GP hybrid conductors as alternative TCE material, their applications in commercial devices are limited due to their low optical transmittance (*TT*) stemming from reduced *TT* (2.3%) per single layer of GP [8,9,10,11,12]. It is required to increase *TT* of hybrid conductors by combining an Ag NW network with a patterned GP mesh with large void areas [11]. 

In order to make the hybrid conductor of Ag NW-GP with high optical and electrical properties, there has been big progress in GP mesh engineering by tailoring its pattern into predefined shapes, position, and sizes at atomic scale mainly through (i) vacuum processes of He-ion/electron beam lithography, O_2_ plasma etching, nanoimprint lithography, catalytic passivation, catalytic etching and hot embossing imprinting [13,14,15,16,17,18,19,20], and (ii) non-vacuum approaches of laser-assisted transfer printing, laser ablation, micro-molding, and masking layer based mesh- patterning [21,22,23,24,25,26,27]. 

However, despite the intense research efforts towards GP patterning methods through various routes [13,14,15,16,17,18,19,20,21,22,23,24,25,26,27,28,29,30,31,32,33], the above GP patterning processes failed to make a patterned GP for commercialized flexible display or electronic devices due to following issues. First, most GP patterning approaches require complicated, low durability and expensive lithographic steps, instruments, and materials, leading to lacked scalability and low production speed of GP meshes [13,14,15,16,17,18,19,20,21,22,23,24,25,26,27,28,29]. Specifically, the as-mentioned patterning process for GP involves multiple steps of complicated template/mask preparation (mask lithography), lifting/stamping of patterned GP onto a substrate, photoresist (PR) developing, etching, PR/mask removal, and laser ablation [13,14,15,16,17,18,19,20,21,22,23,24,25,26,27]. Second, the optical interference effect (i.e., Moire phenomenon) can be observed in the display image because of a constructive and destructive interferences by regularly arrayed voids in GP mesh [34,35]. Third, there can be a constraint by the patterning conditions depending on the target substrate [13,14,15]. To our best knowledge, a cost-effective process for GP patterning and its hybridization remains as the major technological obstacle limiting the large-scale manufacturing of TCEs based on GP mesh hybrid [13,14]. It is required to develop a facile and scalable process for GP mesh based flexible TCE conductor on target substrates with desired conformation. 

Herein, we report a novel approach to prepare the flexible TCE based on a hybrid of irregularly patterned GP mesh and Ag NW networks through the chemical etching-driven patterning without employing vacuum technology-based semiconductor processes. Our idea is to make a patterned GP mesh by the chemical etching of the edge atom on a grain boundary of GP. Specifically, as grain boundary or defects of GP are relatively vulnerable to oxidation/reduction chemicals compared to its basal planes, the island GP along with line defects (grain boundaries) can be easily etched and separated by the chemical etching, resulting in the formation of irregularly etch-patterned GP mesh. Considering the presence of many voids of disconnected GP (GP mesh), it might be logical to expect the superior electro-optical property and bending stability of Ag NW-GP mesh hybrid to that of an Ag NW or the Ag NW-GP hybrid [36]. 

Very different from conventional patterned GP, our GP mesh has following advantages. First, our GP mesh can be prepared on various substrates and scalable to wafer-scale through the low-cost solution process without using the vacuum process-based patterning instruments. Second, the patterned GP mesh structure by chemical etching can be achieved in a short time (<10 min) without requiring extra preparation steps because carbon atoms of the GP films are removed through a reaction between GP and etching radicals. Third, the hybrid conductor of Ag NW-GP mesh exhibits high optical transmittance without the chemical deterioration with time. 

Based on our current approach, we prepared hybrid conductors of Ag NW and GP mesh with high optical transmittance and mechano-electric stability after systematical investigation on their morphological, physico-chemical, and mechanical properties as a function of concentration and treatment-time of chemical etchant. 

## 2. Materials and Methods 

### 2.1. Material Syntheses

Conducting materials including graphene (GP) nanosheet and Ag NW were used as received from makers. Other unspecified reagents were purchased from Sigma-Aldrich (St. Louis, MO, USA) and used without further purification.

**Figure 1:**
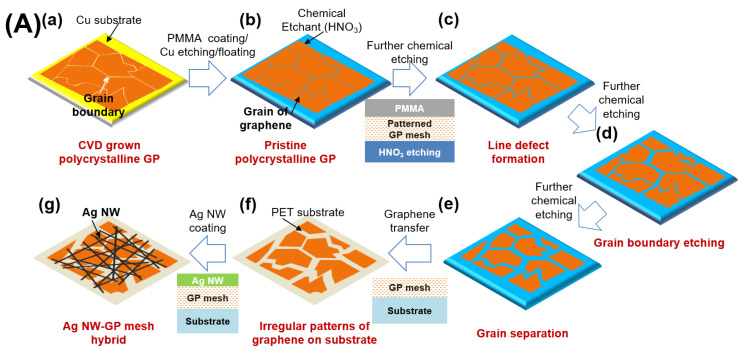

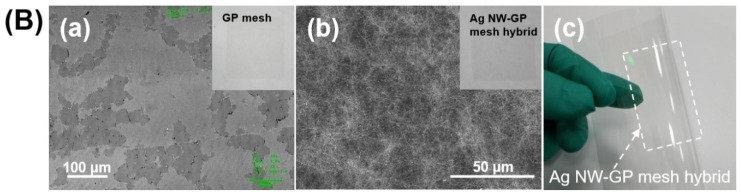
(**A**) schematic illustration for preparation of irregularly patterned GP mesh and its hybrid with Ag NWs (Ag NW-GP mesh hybrid). (**a**) polycrystalline GP prepared by chemical vapor deposition method, (**b**) PMMA coated pristine polycrystalline GP floated on chemical etchant, (**c**) formation of line defect by chemical etchant on the GP, (**d**) more line defects formation leading to the etched grain boundary of GP, (**e**) formation of irregularly patterned GP mesh of separated GP grains, (**f**) as-formed irregularly patterned GP mesh transferred on substrate, (**g**) hybrid conductor film of Ag NW and patterned GP mesh; (**B**) morphology of conductors: (**a**) optical image of irregularly patterned GP mesh (HNO_3_) by OM, (**b**) electron image of an Ag NW hybrid with irregularly patterned GP mesh (HNO_3_) by SEM, (**c**) digital-photo image of transparent conductive film of Ag NW-GP mesh hybrid.

*Preparation of irregularly patterned graphene (GP) mesh*: A schematic of the preparation procedure for the irregularly patterned TCEs is illustrated in Figure 1A. Briefly, as-received chemical vapor deposition (CVD) grown polycrystalline graphene (GP, Graphene Square Inc., Seoul, Korea) on Cu foil was covered with polymethylmethacrylate (PMMA) through spin-coating, followed by removal of Cu substrate by a FeCl_3_-based etchant. After washing the Cu etchant with deionized water, the free-standing polycrystalline GP/PMMA bilayer was subsequently floated onto acidic chemical etchant (aqueous HNO_3_) to form line defects on the GP layer. Further chemical etching caused the grain separation of GP along the boundaries, resulting in an irregularly patterned GP mesh. The GP mesh was then transferred onto a glass or PET substrate, followed by removal of PMMA by acetone washing. 

*Hybrid conductor of Ag NW network and irregularly patterned graphene (GP) mesh:* Networks of randomly distributed Ag NWs (NW length = 20 ± 5 μm; NW diameter = 30 ± 5 nm) were purchased from a commercial maker (Aiden) to be formed onto the GP mesh by one time bar-coating, followed by annealing at 100 °C for 5 min. 

### 2.2. Material Characterizations

For the characterization of the structural, morphological, and surface properties of hybrid conductors, we used an optical microscope (OM, IX71, Olympus, Tokyo, Japan), scanning electron microscope (SEM, Nova 400 Nano SEM, FEI, Hillsboro, OR, USA) at an accelerating voltage of 5 kV, an X-ray photoelectron spectroscopy (XPS, PHI 5000 VersaProbe, ULVAC-PHI, Kanagawa, Japan) with an Al K_α_ (1486.6 eV) source, and a Raman spectrometer (Renishaw, inVia Raman microscope, Gloucestershire, UK) under backscattering geometry with the 633 nm laser at 2.5 mW. We also used a haze meter (NDH 5000, Nippon Denshoku Industries, Tokyo, Japan) and surface resistivity meter (R-CHEK, model RC2175, EDTM, Toledo, OH, USA) to measure the optical transmittance and the sheet resistance of the hybrid conducting films.

### 2.3. Mechano-Electric Characterization

The mechano-electric properties of hybrid conductor Ag NWs and irregularly patterned GP mesh were characterized by measuring the fractional bending-resistance change (Δ*R/R*_o_ (%)) of conductive film with a cyclic folding tester (CFT-200, Covotech, Hwaseong, Korea). In the bending fatigue test, the strain of the conducting films was fixed to 6.7% (bending radius: 1 mm) by setting the PI substrate thickness to 125 μm. The bending-resistance (Δ*R/R*_o_ (%)) of hybrid conductor films was measured and recorded after 40,000 and 200,000 bending cycles at a bending frequency of 1 Hz. 

## 3. Results and Discussion

### 3.1. The Hybrid Conductor of Ag NW and Irregularly Patterned GP Mesh

Hybrid transparent conducting films of silver nanowires (Ag NW) and irregularly patterned graphene (GP) mesh were prepared by bottom-etching of GP followed by hybridization with Ag NWs) networks, as illustrated in Figure 1A [9,11]. First, CVD-grown polycrystalline GP on a copper (Cu) substrate was covered by poly(methyl methacrylate) (PMMA), acting as a carrier and a protection layer during the etching. The Cu layer in the Cu/GP/PMMA multilayer was then removed by FeCl_3_ etchant (Figure 1A: a→b). After Cu removal, the bottom of polycrystalline GP/PMMA bilayer was etched by placing the film on an etchant solution (HNO_3_) with a controlled concentration and time to form irregular patterns on GP (Figure 1A: b→c). The initial line defect patterns were formed beneath on polycrystalline GP/PMMA by the bottom-chemical etching under a relatively low etchant concentration (<1.5 M), inducing separation of grains (Figure 1A: c→d). Further grain separations beneath on GP/PMMA took place under a higher etchant concentration (1.5–5 M) (Figure 1A: d→e). As-isolated grains from others were formed on GP/PMMA after long etching time (>10 min) or under higher etchant concentration (>5 M). That is, collection of isolated grains of GP resulted in patterned GP mesh with irregular patterns. After chemical etching, GP mesh/PMMA was transferred onto a plastic substrate (polyethylene terephthalate; PET) followed by removal of the PMMA layer by acetone washing/rinsing, resulting in GP mesh on a PET substrate (Figure 1A: e→f). Then, the flexible hybrid conductor of Ag NW networks and GP mesh were formed by bar-coating of Ag NW solution on GP mesh/PET (Figure 1A: f→g). The hybrid conductor (Ag NW/GP mesh) films had a high optical transmittance (*TT*: 97.4%; 90.5% including the PET substrate) with a moderate conductivity (sheet resistance, *R*_s_ = 800 Ω/sq.). Note that the *R*_s_ and *TT* can be regulated by judicious control of precursor concentration and etching conditions. 

Figure 1B displays the optical/electronic microscopic or digital photographic images of GP mesh (Figure 1B-a) and its hybrid conductors of Ag NW-GP mesh (Figure 1B-b,c). All insets (Figure 1B-a,b) included in optical microscopic (OM) figures are the corresponding digital images of the conductor films of patterned GP mesh and its hybrid with Ag NW, respectively. Figure 1B-a,b displays the OM image of irregularly patterned GP mesh by HNO_3_ etchant and its hybrid conductor of Ag NW and GP mesh. Note that, although pristine polycrystalline GP before etch-patterning shows a smooth surface with a little line defect yet noticeable splits (Figure S1), GP mesh shows many obviously visible splits from removed grains after etch-patterning (Figure 1B-a). Then, as observed by scanning electron microscopy (SEM), a hybrid conductor of Ag NW and GP mesh (Figure 1B-b) exhibits a clear network of Ag NWs formed on the irregular patterned GP mesh. Figure 1B-c displays the digital photograph of the hybrid conductor of Ag NWs and patterned GP mesh on a plastic substrate (PET), exhibiting high flexibility and optical transparency.

**Figure 2:**
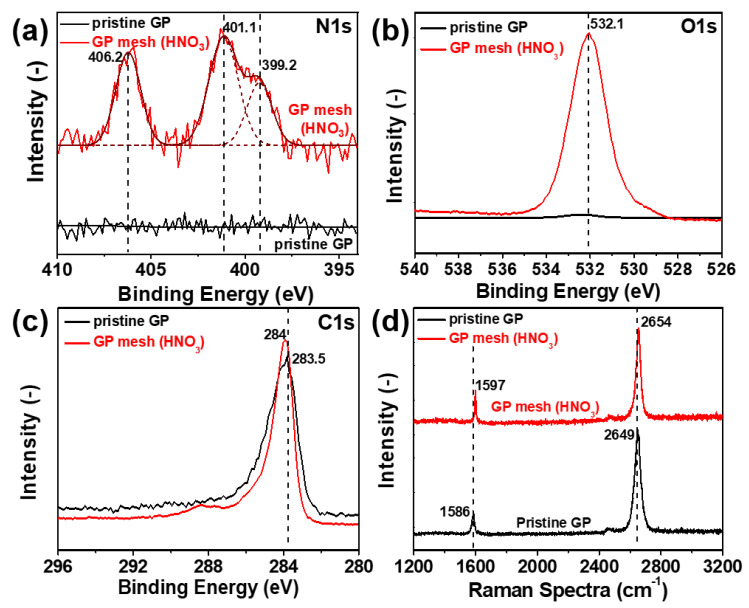
Structural analyses of irregularly patterned GP mesh and pristine GP by chemical etchants (treatment: 5 M HNO_3_ for 10 min) X-ray Photoelectron Spectroscopy (XPS) of pristine and patterned GP meshes by etch-patterning (**a**) N1s, (**b**) O1s, (**c**) C1s; Raman spectroscopy of pristine and GP meshes before and after etch-patterning, (**d**) D and G band of pristine and patterned GP mesh.

Figure 2 compares the X-ray photoelectron (XPS, Figure 2a–c) and Raman spectroscopic (Figure 2d) information of the pristine GP and patterned GP mesh with HNO_3_ etching (10M, 10 min). Figure 2a–c show the XPS results for nitrogen 1s (N1s), oxygen 1s (O1s) and carbon 1s (C1s) signals, respectively. These N1s and O1s peaks were not present in the pristine GP but detected after etching while the C1s signals are present both in pristine GP and patterned GP mesh. In Figure 2a, N1 signals were analyzed by deconvolution of XPS curves through the Gaussian fitting. We found three peaks at 406.2 eV (NO_x_ bond), 401.1 eV (N-H (or pyrrolic bond)), and 399.2 eV (C-N (pyridinic bond)) [9,37,38,39]. As-assigned peaks suggest the chemical doping of GP mesh with etchant (HNO_3_), considering the various nitrogen related bonding formations including NO_x_, pyrrolic, and pyridinic bonds [9,37,38,39]. As displayed in Figure 2b, O1s peaks (532.1 eV) are observed only in GP mesh, indicating the presence of chemical dopant (NO_x_) on the GP mesh. Figure 2c compares the XPS C1s of pristine GP and GP mesh. The shift of the C1 peak position (or binding energy) from pristine GP (283.5 eV) to GP mesh (284 eV) suggests the partial oxidation of carbon by chemical etching with HNO_3_ [3,9]. Figure 2d compares the Raman spectra of the pristine GP and patterned GP mesh to investigate the chemical etching effects on GP mesh. As displayed in the spectra, blue shifts of both G and 2D peaks are observed in patterned GP mesh; the G-band (1586 cm^−1^) in the pristine GP shifted to a new position (1597 cm^−1^) in GP mesh by 11 cm^−1^ by HNO_3_ etching. Such a blue shift (upward shift) of G-band upon etching is attributable to the phonon stiffening by HNO_3_ induced charge extraction [9,39]. In addition, consistent with the XPS results, the Raman shift in the 2D-peak from 2649 to 2654 cm^−1^ for GP mesh is observed due to the nitrogen doping on GP mesh [9,37,38,39]. 

### 3.2. Electro-Optical and Mechano-Electric Characteristics of Conducting Films 

**Figure 3:**
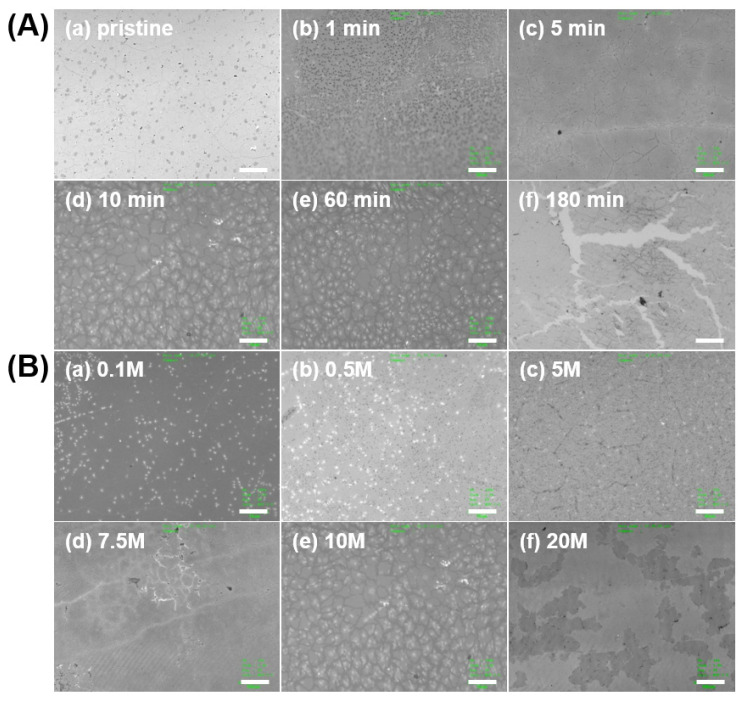
Morphology of irregularly patterned GP meshes obtained by optical microscope. (**A**) effect of etchant treatment time (0–180 min) on the morphology of GP mesh using HNO_3_ (10 M) as chemical etchant (**a**) un-treated (pristine GP), (**b**) 1 min, (**c**) 5 min, (**d**) 10 min, (**e**) 60 min, (**f**) 180 min; (**B**) effect of etchant (HNO_3_) concentrations (0.1–20 M) on the morphology of GP mesh treated for 10 min. (**a**) 0.1 M HNO_3_, (**b**) 0.5 M HNO_3_, (**c**) 5 M HNO_3_, (**d**) 7.5 M HNO_3_, (**e**) 10 M HNO_3_, (**f**) 20 M HNO_3_. (Scale bar: 15 μm).

We systematically investigated the effect of etching condition (etchant concentration and treatment time) on the morphology of GP meshes. Figure 3 shows morphological changes of irregularly patterned GP meshes treated with chemical etchant (HNO_3_) by displaying their optical microscopic images at various treatment time (Figure 3A; 0–180 min) and concentrations (Figure 3B; 0–20 M) of chemical etchant. 

Figure 3A display the optical microscopic (OM) images of patterned GP meshes formed by HNO_3_ (10 M) obtained at various etchant treatment time (0–180 min). In the initial stage of etching (<1 min), there are only dimmed grain boundaries visible in the pristine polycrystalline GP (Figure 3A-a) while formation of many etched points are observed at short treatment time (1 min, Figure 3A-b). After elongated treatment (>5 min), however, many line defects of GP grain boundaries are observed (Figure 3A-c–f). As-formed line defects are intensified with elongated treatment time (>10 min) until severe separation of grains (grain island) and formation of secondary line defects in the grain islands (Figure 3A-f). Note that all the GP meshes show similar pattern morphology under the circumstance of any range of treatment time (>10 min) with the etchant of 10 M HNO_3_. As shown in Figure 3B, various pattern shapes are formed at different concentrations of etchant (0.1–20 M) at fixed treatment time (10 min). Similar to the result of treatment time effect on the morphologies in Figure 3A, there were no noticeable line defects formed at low etchant concentration (<0.5 M; Figure 3B-a,b). However, it begins to show isolated grains (or more intensified grain boundaries) on GP at higher etchant concentration (>5 M), forming irregularly patterned GP meshes (Figure 3A-c–f). We found that isolated grain patterns of GP mesh are formed at higher etchant concentration (>10 M), indicating accelerated defect formations on polycrystalline GP by a higher dose of etchant treatment. 

Overall, considering the relationship between etching condition (concentration and treatment time) and pattern morphologies of GP mesh, the interaction between etching chemicals and grain boundary of GP plays a crucial role to determine the morphology of GP mesh such as the pattern size, etched grain number, and etching direction. Interestingly, thanks to such irregular pattern of GP mesh, our GP mesh does not have superimposed visual interference, a so-called Moiré effect, possibly leading to enhanced image resolution of display devices.

**Figure 4:**
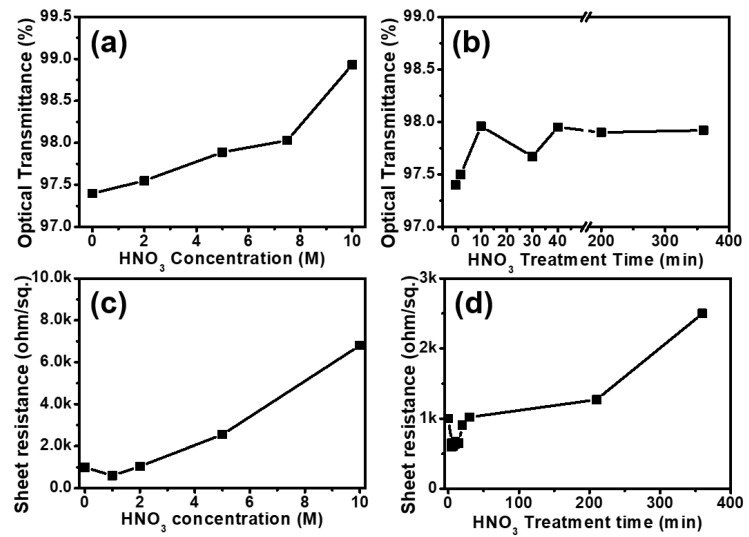
Optical and electrical properties of irregularly patterned GP meshes prepared by chemical etchant (HNO_3_); plot of optical transmittance (%) measured at 550 nm as a function of (**a**) HNO_3_ concentration and (**b**) HNO_3_ treatment time; electrical conductivity (sheet resistance, Ω/sq.) of irregularly patterned GP meshes prepared by chemical etchant (HNO_3_); plot of sheet resistance (Ω/sq.) of irregularly patterned GP meshes as a function of (**c**) etchant (HNO_3_) concentration and (**d**) etchant (HNO_3_) treatment time.

As shown in Figure 4, assuming the enhanced electrical and optical properties of the GP mesh by suitable treatment, we further investigated the effect of etching condition (HNO_3_ concentration and treatment time) on the optical (Figure 4a,b) and electrical properties (Figure 4c,d) of GP meshes. Figure 4a,b display that the optical transmittance (*TT*) of the GP mesh as a function of etchant concentration (Figure 4a) and treatment time (Figure 4b). Figure 4a displays that the *TT* of GP mesh increases linearly with the etchant concentration (0–10 M), suggesting more vacant space (or emptied space among the grains) formation on GP mesh at higher concentration. As shown in Figure 4b, although there is some enhancement of *TT* of GP mesh by the elongated treatment for the short time range (0–10 min), the *TT* of GP meshes do not change much after certain etching time (>40 min). That is, in the comparative optical characterization of GP mesh, we found that the concentrated etchant can more effectively enhance the *TT* of GP mesh than that of the etching time does under constant etchant concentration. We also evaluate the effect of etching concentration (Figure 4c) and treatment time (Figure 4d) on the electrical properties (conductivity) of the GP meshes. As shown in Figure 4c, the sheet resistance (*R*_s_) of GP mesh increased almost proportional to the etchant concentration (0–10 M) owing to the disturbance of charge-transport pathways by GP grain boundaries (or enhanced line defects). However, under low concentration range of HNO_3_ (<2 M) and short treatment time (<30 min), GP mesh shows reduced resistance (enhanced conductivity), attributable to the more HNO_3_ doping effect on the GP mesh rather than disturbed charge transport. Figure 4d displays the electrical conductivity of GP mesh as a function of treatment time, suggesting the increased *R*_s_ of GP meshes treated with HNO_3_ by elongated etching time under same concentration, owing to the disconnected grains under elongated treatment time. 

Overall, in the optical and electrical characterization of GP mesh, the transmittance and conductivity (sheet resistance) of GP meshes can be regulated by etchant concentration and treatment time as such conditions can affect the vacant space formation and grain connections (electrical pathway among grains) of GP meshes. 

**Figure 5:**
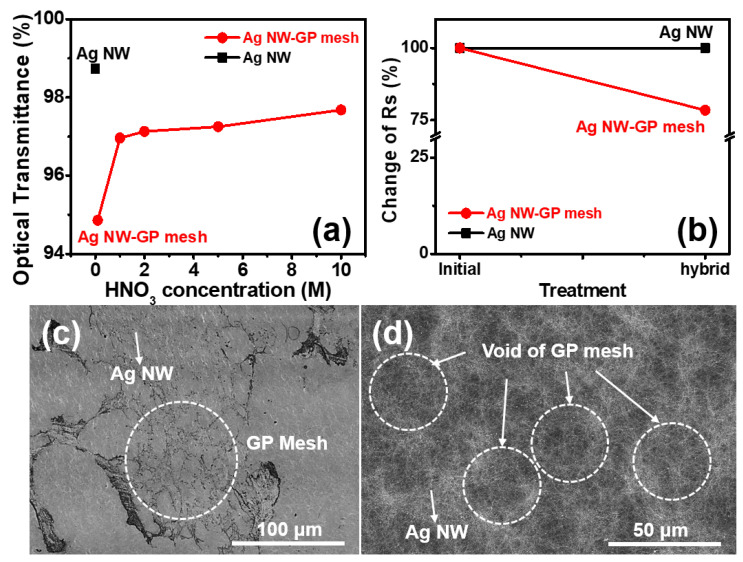
Optical and morphological characterization of Ag NW-GP mesh hybrid. (**a**) optical transmittance and (**b**) electrical conductivity of Ag NW-GP mesh hybrid; morphologies of Ag NW-GP mesh hybrid (**c**) global image of GP mesh in the hybrid by photo-microscopy, (**d**) magnified electronic image of hybrid conductor by SEM.

Figure 5 displays the optical (Figure 5a) and electrical (Figure 5b) properties and morphologies (Figure 5b,c) of the hybrid conductor of Ag NW and GP mesh. As displayed in the *TT* plot of hybrid conductor and Ag NW as a function of etchant concentrations at constant treatment time (10 min) (Figure 5a), the *TT* of hybrid conductor increases proportional to the etchant (HNO_3_) concentration. Such a high dependence of etchant concentration of hybrid on their *TT* is attributable to more formation of voids (or defective vacant spaces) by using higher concentration etchants (HNO_3_), consistent with the result of *TT* vs. etchant concentration of GP meshes (Figure 4a). Figure 5b displays higher electrical conductivity (Δ*R_s_*: 22%) of hybrid conductor than that of control (Ag NW), suggesting the synergic electrical percolating effect of hybrid conductor by convolution of electrical pathways of Ag NW network and GP meshes. Figure 5c,d display the morphologies of hybrid conductors of Ag NW and GP meshes obtained by photo-microscopy (Figure 5c) and SEM (Figure 5d). As shown in dashed circle of microscopic photo-image (Figure 5c), pattern morphology of hybrid conductor is retained without destruction of GP mesh structure after hybridization with Ag NW. In the enlarged image of hybrid conductor by scanning electron microscopy (SEM) (Figure 5d), the void area of GP meshes under Ag NW networks is clearly visible, indicating the retained mesh pattern of GP. 

**Figure 6:**
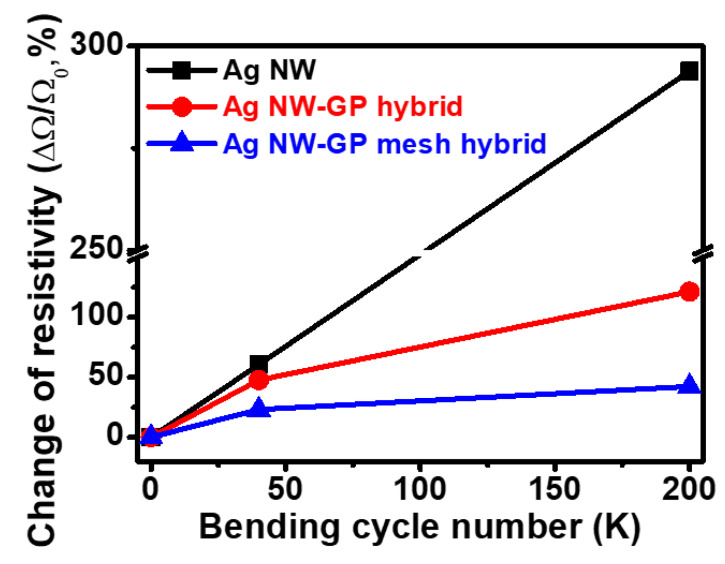
Cyclic bending fatigue test measured at 1R for 200k cycles for conductors (Ag NW, Ag NW- pristine GP hybrid (Ag NW-GP hybrid) and Ag NW-irregularly patterned GP mesh hybrid (Ag NW-GP mesh hybrid).

Figure 6 compares the mechano-electric stability of conductors (Ag NW, Ag NW-GP hybrid and Ag NW-GP mesh hybrid) by measuring the resistivity changes (Δ*R*/*R*_o_) under repeated bending conditions at 1 mm bending radius (1R, 6.7% strain) up to 200,000 (200k) cycles. Before the cyclic bending fatigue test, the electrical conductivities (resistivity) of Ag NW, Ag NW-GP hybrid, and Ag NW-GP mesh hybrid are 134.4-Ω, 128.7-Ω, and 129.5-Ω, respectively. After the cycles, a difference of the linear resistance (Δ*R*) between the initial (*R*_o_, before cycles) and final resistance (*R*_200,000_, after 200,000 cycles) for conductors are measured as a function of bending cycles. Noticeably, our hybrid conductor (Ag NW-GP mesh) exhibits superior bending stability (or reduced resistance change (Δ*R*/*R*_o_) of 42.4% after 200k cycles) to that of control conductors (Ag NW (Δ*R/R*_o_: ~293% at 200k cycles), Ag NW-pristine GP hybrid (Δ*R/R*_o_: ~121% at 200k cycles)) under repetitive bending cycles. Such an enhanced mechano-electric stability of our hybrid can be attributed to (i) combined electronic path formation with Ag NWs and GP mesh, (ii) elastic nature of Ag NW, and (iii) better interaction with Ag NW with substrate by unique mesh structure [9,11,40,41]. 

In the comparison of mechano-electric characteristics, we can observe the enhanced mechano-electric stability of hybrid conductor to Ag NW network owing to provided auxiliary local electron transport pathways by GP layer (pristine and patterned GP). Note that the Ag NW-GP mesh hybrid exhibits superior mechano-electric stability to that of Ag NW-GP hybrid using non-patterned pristine GP, attributable to the alleviated detachment of Ag NW and GP mesh layers. Specifically, filled space between Ag NW and GP mesh thus reduces deformation and resistance of the Ag NW network. That is, considering internal friction induced mechanical instability of hybrid conductor caused by poor adhesion of GP on Ag NWs during the repetitive folding and stretching, void spaces on GP mesh could effectively enhance the mechanical strength of hybrid conductor by preventing sliding/detachment of hybrid conductor from the substrate [11]. 

## 4. Conclusions

In summary, we prepared a hybrid conductor of Ag NW and irregularly patterned graphene (GP) mesh through the simple and scalable processes of chemical etching and solution coating. For the hybrid conductor preparation, the irregularly patterned GP mesh was prepared via the separation of grains in the polycrystalline GP by utilizing the vulnerable nature of grain boundary to acidic chemical etchant (HNO_3_). The hybrid conductor of Ag NW and irregularly patterned GP mesh, then, was constructed on flexible supports (i.e., plastic substrates) through subsequent film transfer and Ag NW hybridizations. 

Our unique structure of Ag NW and GP mesh hybrid endows us with the following advantages as described below:

1. Facile and simplified fabrication process: The hybrid conductor of Ag NW-irregularly patterned GP meshes on various flexible plastic substrates can be prepared via simple and facile chemical etching and solution deposition process without using complicated vacuum technology process.
2. Tunable pattern shape and size: The shape and size of patterned GP mesh can be judiciously regulated by controlling the concentration and treatment time of etchant chemicals.
3. Controllable electrical-optical properties: The optical transmittance and electrical conductivity of the GP mesh can be regulated by the concentration and treatment time of chemical etchant (HNO_3_). Judicious control of etching condition allows the enhanced optical transmittance and controlled conductivity of the hybrid conductor.
4. Prevented Moiré effect: In contrast to conventional GP mesh containing regular array patterns, GP mesh with irregular patterns can prevent the Moiré phenomena, allowing high resolution display image in the flexible TCE.
5. Enhanced mechano-electric stability: A hybrid conductor of Ag NW and GP mesh exhibits enhanced mechano-electric stability (Δ*R*/*R*_o_: 42.4% at 200k cycles) at elongated bending/unbending cycles under 1R curvature (6.7% strain), superior to that of controls (Ag NW and Ag NW-pristine GP hybrid). Such an improved bending stability of our hybrid conductor can be ascribed to (i) combined electronic pathway formation among Ag NWs and GP mesh, (ii) elastic nature of Ag NW, and (iii) enhanced adhesion with Ag NW with substrate caused by GP mesh.

As demonstrated in this report, we believe that our hybrid conductor prepared with chemical etching-solution deposition process can be an effective and practical TCE candidate for flexible electronic devices [8,9,11,41]. Considering various chemical etchants and flexible substrate candidates, the improved physico-chemical property of our conductor and process feasibility can be expected, and it is still underway to further enhance their device performance. 

## Supplementary Materials

The following are available online at https://www.mdpi.com/2072-666X/11/6/578/s1, Figure S1: Digital photo-image of pristine graphene nanosheet.
