# Supplementary material for: Novel Hybrid Conductor of Irregularly Patterned Graphene Mesh and Silver Nanowire Networks
Source: Micromachines (Basel). 2020 Jun 9;11(6):578. doi: 10.3390/mi11060578 (PMC7345882; doi:10.3390/mi11060578)
Supplement: Supplementary file 1 [file micromachines-11-00578-s001.pdf]

micromachines

Novel Hybrid Conductor of Irregularly Patterned Graphene Mesh and Silver Nanowire Networks

Hiesang Sohn, Weon Ho Shin, Dohyeong Seok, Taek Lee, Chulhwan Park, Jong-Min Oh, Se Yun Kim and Anusorn Seubsai

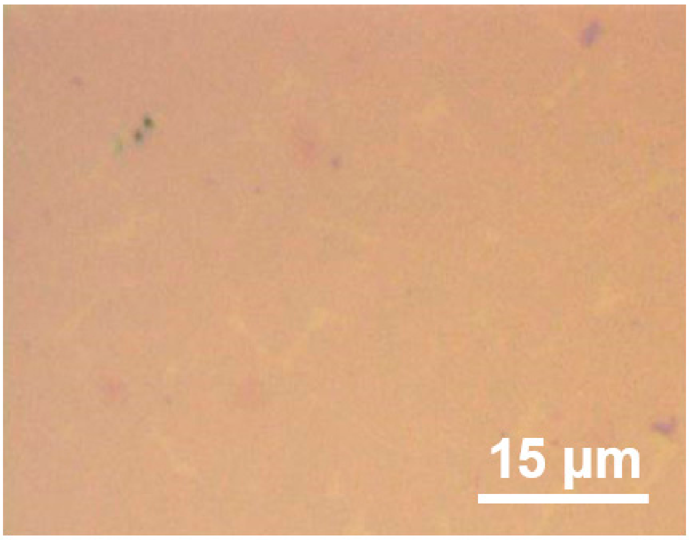

Figure S1. Digital photo-image of pristine graphene nanosheet.
